# Activation of the Interferon Induction Cascade by Influenza A Viruses Requires Viral RNA Synthesis and Nuclear Export

**DOI:** 10.1128/JVI.03109-13

**Published:** 2014-04

**Authors:** Marian J. Killip, Matt Smith, David Jackson, Richard E. Randall

**Affiliations:** School of Biology, Biomedical Sciences Research Complex, North Haugh, University of St. Andrews, St. Andrews, Fife, United Kingdom

## Abstract

We have examined the requirements for virus transcription and replication and thus the roles of input and progeny genomes in the generation of interferon (IFN)-inducing pathogen-associated molecular patterns (PAMPs) by influenza A viruses using inhibitors of these processes. Using IFN regulatory factor 3 (IRF3) phosphorylation as a marker of activation of the IFN induction cascade that occurs upstream of the IFN-β promoter, we demonstrate strong activation of the IFN induction cascade in A549 cells infected with a variety of influenza A viruses in the presence of cycloheximide or nucleoprotein (NP) small interfering RNA (siRNA), which inhibits viral protein synthesis and thus complementary ribonucleoprotein (cRNP) and progeny viral RNP (vRNP) synthesis. In contrast, activation of the IFN induction cascade by influenza viruses was very effectively abrogated by treatment with actinomycin D and other transcription inhibitors, which correlated with the inhibition of the synthesis of all viral RNA species. Furthermore, 5,6-dichloro-1-β-d-ribofuranosyl-benzimidazole, an inhibitor that prevents viral RNA export from the nucleus, was also a potent inhibitor of IRF3 activation; thus, both viral RNA synthesis and nuclear export are required for IFN induction by influenza A viruses. While the exact nature of the viral PAMPs remains to be determined, our data suggest that in this experimental system the major influenza A virus PAMPs are distinct from those of incoming genomes or progeny vRNPs.

**IMPORTANCE** The host interferon system exerts an extremely potent antiviral response that efficiently restricts virus replication and spread; the interferon response can thus dictate the outcome of a virus infection, and it is therefore important to understand how viruses induce interferon. Both input and progeny genomes have been linked to interferon induction by influenza viruses. However, our experiments in tissue culture cells show that viral RNA synthesis and nuclear export are required to activate this response. Furthermore, the interferon induction cascade is activated under conditions in which the synthesis of progeny genomes is inhibited. Therefore, in tissue culture cells, input and progeny genomes are not the predominant inducers of interferon generated by influenza A viruses; the major viral interferon inducer(s) still remains to be identified.

## INTRODUCTION

The genome of influenza A viruses (IAVs) is carried by eight segments of negative-sense viral RNA (vRNA). Each vRNA segment is separately packaged into viral ribonucleoprotein (vRNP), in which it is associated with viral nucleoprotein (NP) and the viral polymerase complex. These vRNPs form helical structures in which the 3′ and 5′ termini are held in a closed conformation by the viral polymerase, while the remainder of the length of the RNA is encapsidated by multiple monomers of NP (reviewed in reference 1). The viral polymerase is responsible for both the transcription and replication of vRNPs, which occur in the nuclei of infected cells, unlike most other RNA viruses. The polymerase generates viral mRNA transcripts using vRNPs as a template; this process requires the polymerase cap-snatching activity to cleave 10- to 15-nucleotide (nt) RNA fragments from the 5′ ends of cellular pre-mRNAs that function as primers for viral mRNA synthesis. In addition, a polyuridine stretch at the 5′ end of each genome segment directs the polyadenylation of viral transcripts. To replicate the virus genome, the viral polymerase first synthesizes cRNA, the full-length complement of vRNA, which is packaged into complementary RNPs (cRNPs); cRNPs function as a replicative intermediate that directs the synthesis of large numbers of progeny vRNPs, from which secondary transcription can occur prior to their nuclear export and assembly into progeny virions.

The interferons (IFNs) are a family of cytokines produced in response to virus infection that can potentially exert powerful antiviral effects through the upregulation of many different interferon-stimulated genes (ISGs) in both infected cells and neighboring uninfected cells. Thus, the IFN response very effectively restricts virus replication and spread (reviewed in reference 2), and as a result, most viruses encode antagonists of this response. For influenza viruses, the principal IFN antagonist is the small multifunctional NS1 protein that targets the IFN system at multiple stages (reviewed in references 3 and 4). IFN is induced during RNA virus infections through the detection of viral pathogen-associated molecular patterns (PAMPs) by cellular pathogen recognition receptors (PRRs). The principal PRRs involved in detecting RNA virus infections are the cytosolic RNA helicases RIG-I and MDA-5; PAMP binding to these receptors activates a downstream signaling pathway that culminates in the activation and nuclear translocation of the ATF2/c-Jun, NF-κB, and IFN regulatory factor 3 (IRF3) transcription factors and subsequent transcription from the IFN-β promoter. IFN induction by influenza A viruses is thought to occur primarily through RIG-I activation (5–7). Much previous work has been carried out in order to characterize the RNA ligands capable of activating RIG-I *in vitro*; it is now generally thought that RIG-I activation requires short blunt-ended double-stranded RNA (dsRNA) with a free 5′ppp group (reviewed in reference 8), although single-stranded RNA with complementary termini that can form panhandle structures containing small stretches of dsRNA are also able to activate RIG-I (9, 10). It has therefore been suggested that base pairing between the partially complementary 3′ and 5′ termini of the influenza virus genome is responsible for RIG-I activation by this virus (7, 11), although the requirement for bound viral polymerase in the maintenance of this base pairing (12) may preclude RIG-I binding to the genome termini. The nature of the viral RNA that associates with RIG-I during influenza virus infections has previously been examined by deep sequencing (7); in that study, RIG-I was found to associate with sequences mapping to subgenomic viral RNAs (associated with defective viruses) and the shorter genome segments. However, since all RNA species that immunoprecipitated with RIG-I were sequenced in this analysis, no distinction was made as to whether these RNAs were vRNA, cRNA, or mRNA. The relative contributions of virus replication and transcription to IFN induction by influenza virus were examined in another study using vRNP reconstitution experiments (11); here, it was reported that IFN induction required progeny vRNA synthesis but not virus transcription. The ability of progeny genomes to induce IFN has not been demonstrated within infected cells, however, and it therefore remains unclear whether input or progeny genomes or indeed another RNA species generated during infection is predominantly responsible for IFN induction during virus infection. In this regard, it is possible that several different types of PAMP are generated or exposed in infected cells, and their relative contributions to overall IFN induction during a virus infection may vary considerably.

In this study, we have examined the effects of several inhibitors of viral polymerase replication and/or transcription activities on activation of the IFN induction cascade in A549 cells and correlated this with the effects of these inhibitors on viral RNA synthesis; this analysis permitted us to determine the contributions of input and progeny virus genomes to IFN induction within infected cells. We demonstrate that for several different influenza viruses, strong activation of the IFN induction cascade can occur under conditions in which viral replication is inhibited. We also demonstrate that input vRNPs do not function as PAMPs since activation of the IFN induction cascade was sensitive to drugs that inhibited both viral transcription and replication. Thus, a major PAMP responsible for IFN induction by influenza A viruses in this experimental system requires viral RNA synthesis, but not genome replication, for its generation.

## MATERIALS AND METHODS

### Cells and inhibitors.

A549, MRC-5, and MDCK cells (all from the European Collection of Cell Cultures [ECACC]) and their derivatives were grown as monolayers in Dulbecco's modified Eagle's medium (DMEM) supplemented with 10% fetal bovine serum (FBS) at 37°C. Cycloheximide (CHX; used at 50 μg/ml), actinomycin D (ActD; used at 1 μg/ml), or 5,6-dichloro-1-β-d-ribofuranosyl-benzimidazole (DRB; 250 μM) was added to the medium at the time of infection, as indicated. Alpha-amanitin was used at 20 μg/ml and required a 1-h period of pretreatment before virus infection in order to efficiently inhibit viral protein synthesis (data not shown). Poly(I·C) transfections were carried out as described elsewhere (13). Small interfering RNA (siRNA) transfections were carried out using DharmaFECT (Thermo Scientific) according to the manufacturer's instructions. The sequences of NP-1496 and NP-231 siRNAs and their effects on influenza virus replication have been described previously (14) and were synthesized by Thermo Scientific. ON-TARGETplus Non-Targeting Pool (Thermo Scientific) was used as nontarget control siRNA.

### Virus infections.

The influenza A virus stocks of A/Udorn/72 (H3N2) (Ud), A/PR/8/34 (H1N1) (PR8), and A/WSN/33 (H1N1) (WSN) were generated by a low multiplicity of infection (MOI; 0.001 PFU/cell) in MDCK cells in serum-free DMEM supplemented with 2 μg/ml N-acetyl trypsin (Sigma) at 37°C. Viruses were titrated on MDCK cells as previously described (15). Recombinant NS1 mutant viruses Ud-Δ99 (15) and PR8-ΔNS1 (16) were grown and titrated in IFN-defective MDCK cells expressing PIV5/V and BVDV/NPro (17, 18) or PR8/NS1 and BVDV/NPro, respectively. PIV5-VΔC vM2 was generated as previously described (19, 20), and Sendai virus (SeV) Cantell was purchased from Charles River Laboratories. Virus infections were carried out at the indicated multiplicities of infection in serum-free DMEM in the absence of trypsin.

### Immunoblotting and immunofluorescence.

The procedures for immunoblotting and immunofluorescence have been described previously (21, 22). Antibodies used for immunoblotting in this study included monoclonal antibodies raised against phospho-IRF3 (Cell Signaling Technology) and β-actin (Sigma) and polyclonal antibodies against total IRF3 (Santa Cruz) and ISG56 (Santa Cruz). Influenza virus NP and M1 proteins were detected using sheep antisera raised against purified and disrupted X31 virus, while NS1 was detected using purified antisera produced against PR8 NS1 (both antisera produced by Diagnostics Scotland). In immunofluorescence studies, anti-IRF3 (Santa Cruz) and anti-p65 (Cell Signaling) antibodies were used. Immunofluorescence was examined using a Zeiss LSM 5 Exciter confocal microscope.

### *In situ* hybridization.

Probes against NP mRNA for *in situ* hybridization were generated from linearized pcDNA-NP, a plasmid containing a cDNA copy of PR8 segment 5 (23) (a kind gift from P. Digard, University of Edinburgh), using a digoxigenin (DIG) RNA labeling kit (Roche) according to the manufacturer's instructions. A549 monolayers (seeded onto poly-l-lysine-treated coverslips) were infected and/or treated with drugs as indicated. At the desired time postinfection, coverslips were washed with phosphate-buffered saline (PBS), fixed in PBS–5% formaldehyde for 15 min, and then washed twice in PBS for 5 min. Cells were treated with 2 μg/ml proteinase K (in 20 mM Tris [pH 7.5]–2 mM CaCl_2_) at 37°C for 10 min and then rinsed twice in PBS–0.1% Tween 20. Cells were postfixed for 15 min at room temperature (RT) in PBS–5% formaldehyde followed by permeabilization in PBS–0.5% Triton X-100 supplemented with 2 mM vanadyl ribonucleoside complexes (Sigma) for 15 min at RT with gentle shaking. Coverslips were washed three times in PBS for 5 min at RT followed by incubation with a hybridization mixture (50% deionized formamide, 1× Tris-EDTA [TE], 300 mM NaCl, 1× Denhardt's solution, 1 mM dithiothreitol [DTT], 1 U/μl RNasin, 5% dextran sulfate [wt/vol], 500 μg/ml tRNA, 200 μg/ml salmon sperm DNA) for 1 h at 55°C. During this period, labeled probe (100 ng per coverslip) in the hybridization mixture was heated to 95°C for 5 min, followed by quenching on ice for 10 min. Coverslips were incubated overnight with this probe-hybridization mixture at 55°C in a humidified chamber. Following hybridization, coverslips were sequentially washed with 2× SSC–10 mM Tris (pH 7.5) (15 min at RT) (1× SSC is 0.15 M NaCl plus 0.015 M sodium citrate), 0.1× SSC–10 mM Tris (pH 7.5) (15 min at RT), 30% deionized formamide–0.1× SSC–10 mM Tris (pH 7.5) (30 min at 55°C), and 0.1× SSC–10 mM Tris (pH 7.5) (5 min at RT). Bound probe was detected using alkaline phosphatase-conjugated anti-DIG antibody (Roche) and Fast Red tablets (Roche). Fast Red fluorescence was examined with a Zeiss LSM 5 Exciter confocal microscope.

### Primer extension analysis.

RNA was extracted from infected cells using TRIzol (Invitrogen), and the levels of vRNA, cRNA, and mRNA were examined by primer extension analyses as described previously (24). [γ-^32^P]ATP (PerkinElmer) was used to radioactively label oligonucleotides (IDT) complementary to positive- and negative-sense PR8 viral RNA transcripts specific for the NP segment using T4 polynucleotide kinase (Thermoscientific) according to the manufacturer's instructions (5′-CGTTCTCCATCAGTCTCCATCTGTTCG-3′ and 5′-GATGTGTCTTTCCAGGGGCGGG-3′, respectively). A further primer complementary to cellular 5S RNA was included to demonstrate equal loading (25). Labeled oligonucleotides were annealed to total cellular RNA isolated as described and subsequently reverse transcribed to labeled cDNA using Superscript II at 45°C (Life Technologies). To terminate this reaction, the contents were mixed 1:1 with formamide loading buffer and boiled. The samples were then loaded onto a denaturing polyacrylamide gel (7 M urea–6% 19:1 polyacrylamide:bis acrylamide) and run in 1× Tris-borate-EDTA (TBE) buffer. Transcript levels were determined by autoradiography and read on a Fujifilm FLA5000 phosphorimager.

## RESULTS

### Activation of the IFN induction cascade by influenza A viruses can occur when virus protein synthesis and the generation of progeny vRNPs are inhibited.

**FIG 1:**
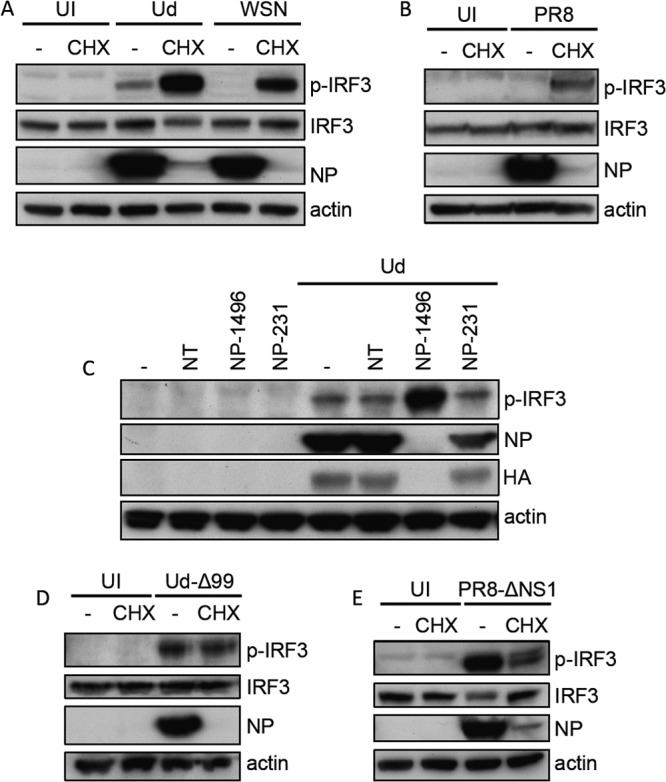
IRF3 activation by influenza A viruses in the absence of virus protein synthesis. (A and B) A549 monolayers were infected with Ud (A), WSN (A), or PR8 (B) at 5 PFU/cell or left uninfected (UI) for 8 h in the presence or absence of CHX. Cell lysates were prepared and subjected to SDS-PAGE and immunoblotting for phospho-IRF3 (p-IRF3), total IRF3, viral NP, and actin. Note that input NP could be detected under CHX treatment conditions upon longer exposures. (C) A549 cells were transfected with no siRNA (−) or 25 mM nontarget (NT) or NP-specific NP-1496 or NP-231 siRNAs for 24 h. Cells were infected with Ud (5 PFU/cell) for a further 8 h, and then cell lysates were prepared and subjected to SDS-PAGE and immunoblotting for phospho-IRF3 (p-IRF3), total IRF3, viral NP and HA, and actin. Note that only NP-1496 siRNA efficiently inhibited NP expression in A549 cells. (D and E) A549 monolayers were infected with Ud-Δ99 (D) or PR8-ΔNS1 (E) at 5 PFU/cell or left uninfected for 8 h in the presence or absence of CHX, and lysates were treated as described above.

First, the requirement for virus protein synthesis and genome replication in the activation of the IFN induction cascade by influenza A viruses was examined. *De novo* NP synthesis and concurrent assembly of nucleocapsids are required for replication of the IAV genome; consequently, by blocking NP synthesis, the translational elongation inhibitor cycloheximide (CHX) prevents viral replication by inhibiting cRNP and progeny vRNP generation and thus secondary transcription (24, 26–29) (see Fig. 6A). A549 cells were infected with several different influenza A viruses in the presence or absence of CHX and cell lysates were probed for the presence of the active, phosphorylated form of IRF3 as a marker of activation of the IFN induction cascade. IRF3 activation occurs prior to transcription from the IFN-β promoter and can thus be used to detect activation of the IFN induction cascade under conditions where cellular protein synthesis is blocked. The phosphorylated form of IRF3 was barely detectable following infection with the influenza virus strains A/WSN/33 (H1N1) (WSN) and A/PR/8/34 (H1N1) (PR8) in untreated cells, while A/Udorn/72 (H3N2) (Ud) induced small amounts of IRF3 phosphorylation (Fig. 1A and B). In contrast, infection with all three of these virus strains resulted in considerable IRF3 activation under CHX treatment conditions in which virus protein synthesis was very efficiently inhibited. To confirm that IRF3 activation occurred in the absence of virus protein synthesis, we used an siRNA approach to inhibit viral NP expression. This has previously been shown to inhibit cRNA and vRNA synthesis and indirectly leads to inhibition of the synthesis of all virus proteins by abrogating secondary transcription (14) (Fig. 1C). Treatment of cells with NP-1496 siRNA led to a very efficient knockdown of NP protein synthesis and also inhibited the expression of other viral proteins, as indicated by a lack of hemagglutinin (HA) synthesis in NP-1496-treated cells (Fig. 1C). This efficient knockdown of viral protein synthesis correlated with a clear increase in IRF3 activation by Ud virus compared to untreated cells, consistent with the results obtained using CHX (Fig. 1A). IRF3 activation was not observed following siRNA-mediated knockdown of NP expression from a transfected plasmid (data not shown), so this effect was specific to the knockdown of NP expression from infecting virus. In contrast, an additional NP siRNA, NP-231, was unable to effectively inhibit NP and HA synthesis under these treatment conditions; this siRNA subsequently had no effect on IRF3 activation by Ud virus. Together, these results suggest that virus protein synthesis and thus the synthesis of progeny vRNPs are not essential for activation of the IFN induction cascade by influenza viruses; indeed, IRF3 activation is actually considerably enhanced under conditions (e.g., CHX treatment and NP siRNA knockdown) in which these processes are inhibited.

We hypothesized that the higher levels of IRF3 phosphorylation in CHX-treated cells than in untreated cells infected with influenza A viruses was due to inhibition of expression of NS1 by CHX and thus alleviation of its IFN-inhibitory effects. To address this, we examined IRF3 activation in the presence of CHX by two viruses that lack a fully functional NS1 protein and are thus unable to effectively antagonize IFN induction: Ud-Δ99, a recombinant Ud virus that lacks most of the effector domain of NS1 (15), and PR8-ΔNS1, in which the entire NS1 gene has been deleted (16). As predicted, both Ud-Δ99 (Fig. 1D) and PR8-ΔNS1 (Fig. 1E) induced considerable IRF3 phosphorylation in untreated cells, consistent with the loss of NS1 function leading to enhanced activation of the IFN response compared to the parental wild-type (wt) viruses. Similar levels of IRF3 phosphorylation were detected in Ud-Δ99-infected cells in the presence or the absence of CHX. This is in contrast to the obvious increase in IRF3 activation seen in Ud-wt-infected cells upon CHX treatment. Thus, the very weak activation of IRF3 during wt infections is most likely due to NS1 inhibiting activation of the IFN induction cascade. That we observe no reduction in IRF3 activation by Ud-Δ99 in cells treated with CHX compared to untreated cells strongly suggests that under these conditions progeny vRNA genomes are not responsible for activating the IFN induction cascade. In contrast to the IRF3 activation profile seen with Ud-Δ99, CHX treatment reduced the levels of IRF3 activation by PR8-ΔNS1 compared to those seen in untreated infected cells. This was not necessarily indicative of an absolute requirement for virus replication in order to activate the IFN induction cascade, however, since IRF3 activation could still be detected under CHX treatment conditions for both PR8-ΔNS1 and the parental PR8 virus (Fig. 1B and E). Furthermore, increasing the multiplicity of infection for PR8-ΔNS1 considerably increased the levels of IRF3 activation in the presence of CHX relative to the amount in untreated infected cells (data not shown; see also Fig. 3E).

**FIG 2:**
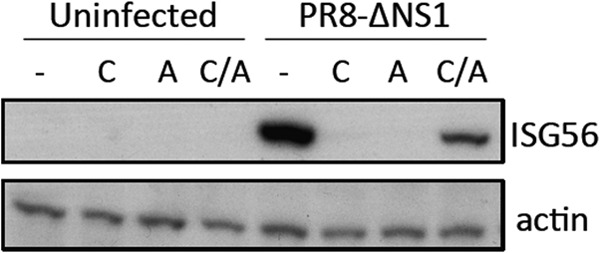
Influenza A viruses induce transcription from IRF3-responsive genes in the absence of virus protein synthesis. A549 cells were infected with PR8-ΔNS1 as indicated in the presence of CHX. At 8 h postinfection (p.i.), monolayers were washed in ActD-containing medium and incubated in the presence of ActD for a further 4 h (C/A). As controls, cells were left untreated (−) or were incubated for 12 h in CHX (C) or ActD (A). Cells were harvested at 12 h p.i. Cell lysates were prepared and subjected to SDS-PAGE and immunoblotting for ISG56 and actin.

To determine whether activation of IRF3 by influenza A viruses in the absence of virus replication also correlated with the transcription of IRF3-responsive genes, a CHX reversal experiment was performed. Cells were infected in the presence of CHX (during which time protein synthesis is inhibited but the accumulation of mRNA transcripts is unaffected) and then reversed in the presence of actinomycin D (ActD) (which forms stable complexes with DNA to inhibit DNA-dependent RNA synthesis); under these conditions, transcripts that have accumulated in the presence of CHX can be translated, while ActD prevents further cellular transcription. IRF3-responsive gene products expressed under these CHX reversal conditions must therefore have been synthesized from transcripts that accumulated in the presence of CHX treatment (and therefore in the absence of viral protein synthesis and genome replication). In accordance with the IRF3 activation by influenza A viruses observed in the presence of CHX (Fig. 1), we could detect expression of the IRF3-responsive gene product ISG56 under CHX reversal conditions (Fig. 2). Clearly, then, activation of the IFN induction cascade and transcription from IRF3-responsive antiviral genes can occur in A549 cells in the absence of virus protein synthesis and therefore in the absence of progeny vRNP generation.

### Actinomycin D inhibits activation of the IFN induction cascade by influenza A viruses.

**FIG 3:**
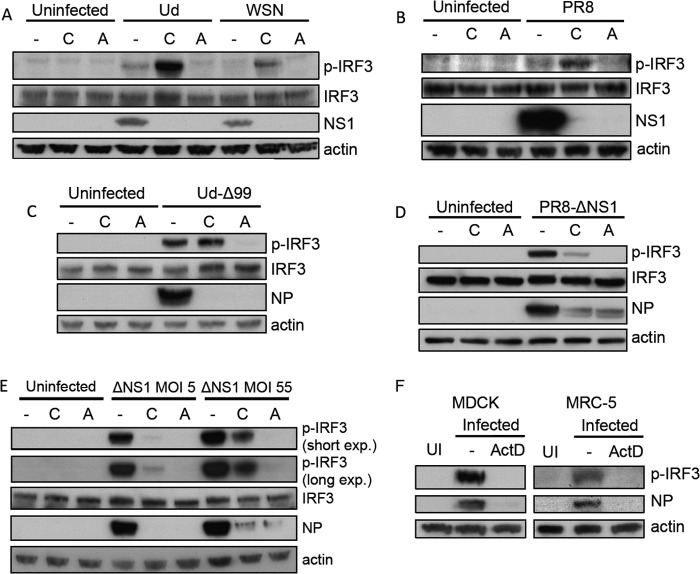
Activation of IRF3 by influenza A viruses is sensitive to actinomycin D. A549 cells were infected with Ud (A), WSN (A), PR8 (B), Ud-Δ99 (C), or PR8-ΔNS1 (D) at 5PFU/cell or left uninfected for 8 h in the presence CHX, ActD, or no drug. Cell lysates were prepared and subjected to SDS-PAGE and immunoblotting for phospho-IRF3 (p-IRF3), total IRF3, viral proteins, and actin. (E) A549 cells were infected with PR8-ΔNS1 at 5 PFU/cell or 55 PFU/cell. At 8 h p.i., cell lysates were prepared and treated as above. exp., exposure. (F) MDCK or MRC-5 cells were uninfected (UI) or infected with 5 PFU/cell of PR8-ΔNS1 in the presence of ActD. At 8 h p.i., cell lysates were prepared and treated as above.

The data presented above indicated that progeny vRNPs are not required for activation of the IFN induction cascade by influenza A virus, but it remained unclear whether input vRNPs were sufficient to induce IFN. While CHX impairs cRNA and vRNA synthesis by the viral polymerase, it does not prevent accumulation of viral mRNA transcripts, although secondary viral transcription is impaired by CHX due to a reduction in available vRNA templates (24) (see Fig. 6A). Influenza A virus transcription by the viral polymerase requires cellular pre-mRNAs to provide 5′-capped primers for transcription initiation (30, 31); consequently, influenza A virus replication is sensitive to inhibitors of cellular transcription (28, 32). Thus, following treatment with ActD, primary viral transcription is blocked and no viral mRNA can be detected in infected cells (33, 34) (see Fig. 6). To determine whether the input virus genome alone was a major activator of the IFN induction cascade or whether viral polymerase activity was required for IFN induction, we examined IRF3 activation in A549 cells treated with ActD. Strikingly, IRF3 activation could not be detected following infection with any of our panel of influenza viruses (Ud, Ud-Δ99, WSN, PR8, and PR8-ΔNS1) in the presence of ActD (Fig. 3). This was a stark difference from the situation following CHX treatment (which often considerably enhanced IRF3 activation), despite both ActD and CHX efficiently inhibiting the expression of viral proteins, including NS1. This inhibition of IRF3 activation by ActD was observed even when the multiplicity of infection was considerably increased; while ∼50 PFU/cell PR8-ΔNS1 induced substantial levels of phospho-IRF3 in untreated and CHX-treated A549 cells, negligible IRF3 activation was detectable in ActD-treated infected cells (Fig. 3E). This effect of ActD was not cell-type specific, since ActD also inhibited influenza-virus-induced IRF3 activation in MDCK and untransformed lung fibroblast MRC-5 cells (Fig. 3F).

**FIG 4:**
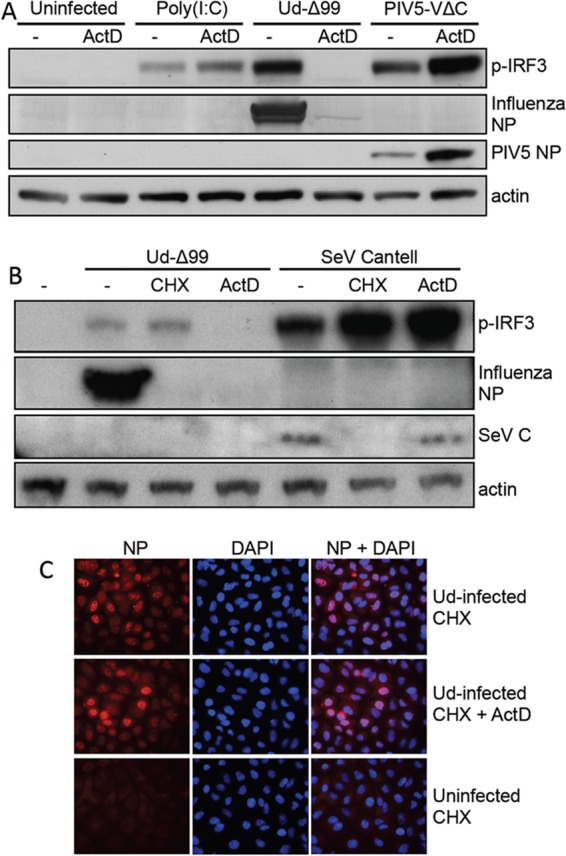
Actinomycin D-mediated inhibition of IRF3 activation is specific to influenza virus infections. (A) A549 cells were transfected with poly(I·C) or infected with Ud-Δ99 or PIV5-VΔC vM2 at 5 PFU/cell in the presence or absence of ActD. After 8 h, cell lysates were prepared and subjected to SDS-PAGE and immunoblotting for phospho-IRF3 (p-IRF3), viral proteins, and actin. Note that the increase in PIV5 NP expression in ActD treatment conditions is due to inhibition of expression of cellular ISGs, likely ISG56, which is a potent inhibitor of PIV5 protein synthesis (68). (B) A549 cells were infected with 5 PFU/cell of Ud-Δ99 or Sendai virus (SeV) Cantell in the presence or absence of CHX or ActD. Cell lysates were prepared at 8 h p.i., subjected to SDS-PAGE, and immunoblotted for p-IRF3, viral proteins, and actin. (C) A549 monolayers were uninfected or infected with Ud in the presence of CHX and ActD or CHX alone. Two hours later, cells were fixed and input vRNPs were detected using antibody to influenza virus NP. Nuclear material was stained with DAPI (4′,6-diamidino-2-phenylindole). NP and DAPI staining was visualized by fluorescence microscopy.

Inhibition of influenza-virus-induced IRF3 activation by ActD was not due to nonspecific effects of the drug on the activation of the IFN induction cascade, since, importantly, ActD did not inhibit IRF3 activation by the synthetic dsRNA analogue poly(I·C) or by PIV5-VΔC vM2 (35), a preparation of parainfluenza virus 5 (a virus insensitive to cellular transcription inhibitors [36]) that is an efficient IFN inducer due to the presence of defective viruses (Fig. 4A). Furthermore, ActD had no effect on IRF3 activation by the Cantell stock of Sendai virus (Fig. 4B), a defective virus-rich preparation that is well characterized as a RIG-I activator (7, 37, 38), indicating that ActD does not inhibit RIG-I activation in general but that its effects are specific to influenza virus. In addition, the inability of influenza A virus to activate IRF3 in ActD-treated cells was not due to impairment of virus entry and RNP import to the nucleus by this drug, since the nuclear localization of input vRNPs was unaffected by ActD treatment (Fig. 4C).

**FIG 5:**
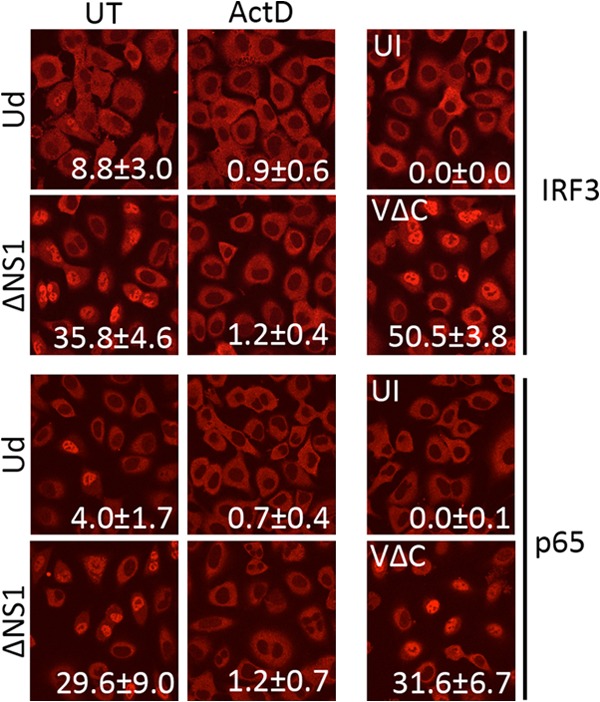
Inhibition of influenza virus-induced IRF3 and p65 nuclear translocation by ActD. A549 cell monolayers were infected with Ud or PR8-ΔNS1 at 5 PFU/cell for 8 h in the presence of ActD. Uninfected cells and cells infected with PIV5-VΔC vM2 (5 PFU/cell) were included as controls. Cells were then fixed and immunostained using antibodies to IRF3 and p65. The nuclear localization of these transcription factors was examined by fluorescence confocal microscopy. The mean percentage of cells (±1 standard deviation [SD]) exhibiting nuclear IRF3 or p65 localization was quantified by scoring nuclear localization from 10 fields of view from each condition; this is indicated on the corresponding image for each condition.

As an additional measure of activation of the IFN induction cascade, we examined the effects of ActD on the nuclear translocation of both IRF3 and the p65 subunit of NF-ΚB, a step that occurs following activation of these transcription factors in the cytoplasm. A549 cells were infected with Ud or PR8-ΔNS1 in the presence of ActD or no drug for 8 h, after which time cell monolayers were fixed and stained for IRF3 and p65 localization. The resulting confocal microscopy images are shown in Fig. 5, and the percentage of cells exhibiting nuclear localization of these transcription factors under each treatment/infection condition is indicated. In the absence of drug, nuclear IRF3 was detectable in 8.8% and 35.8% of cells following infection with Ud or PR8-ΔNS1, respectively. Similar levels of p65 nuclear translocation were observed: 4.0% of cells for Ud and 29.6% of cells for PR8-ΔNS1. Following infection in the presence of ActD, however, the level of nuclear translocation for both IRF3 and p65 dropped to around 1% for both viruses, indicating that ActD efficiently inhibits activation and subsequent nuclear translocation of both of these transcription factors.

### Activation of the IFN induction cascade by influenza A virus requires viral RNA synthesis and nuclear export.

**FIG 6:**
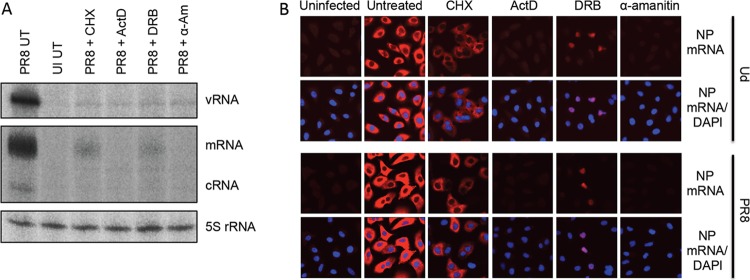
Viral RNA synthesis profiles in the presence of cycloheximide, actinomycin D, 5,6-dichloro-1-β-d-ribofuranosyl-benzimidazole, and α-amanitin. (A) A549 monolayers were infected with PR8 at 5 PFU/cell in the presence of CHX, ActD, DRB, α-amanitin (α-Am; following a 1-h pretreatment of cells), or no drug as indicated. Eight hours later, cells were harvested, and extracted RNA was subjected to primer extension analysis using ^32^P-labeled probes specific for PR8 NP cRNA, vRNA, and mRNA. A further primer complementary to cellular 5S RNA was included as a loading control. (B) A549 cells were infected with Ud or PR8 at 5 PFU/cell in the presence of CHX, ActD, DRB, α-amanitin (α-Am; following a 1-h pretreatment of cells), or no drug as indicated for 8 h. Cells were then fixed, and viral NP mRNA was visualized by *in situ* hybridization (red). Cell nuclei were stained with DAPI (blue).

**FIG 7:**
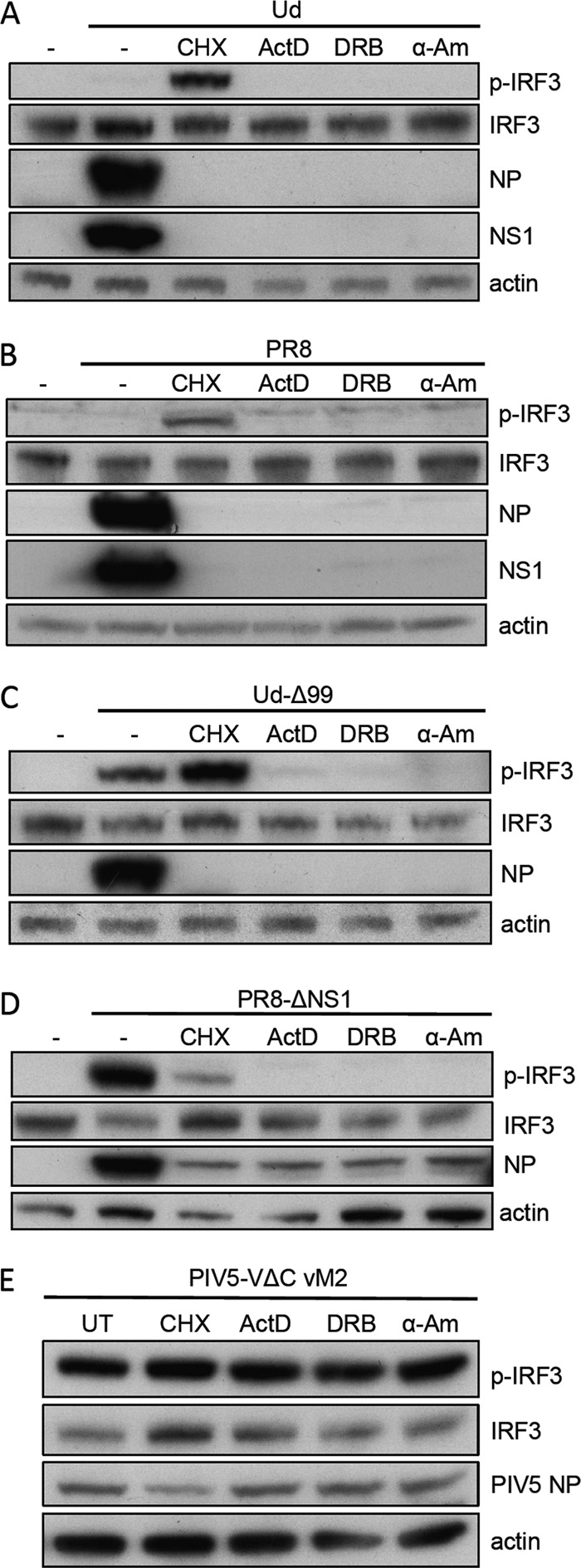
Inhibition of influenza-induced IRF3 activation by actinomycin D, 5,6-dichloro-1-β-d-ribofuranosyl-benzimidazole, and α-amanitin. A549 cells were infected with Ud (A), PR8 (B), Ud-Δ99 (C), PR8-ΔNS1 (D), or PIV5-VΔC vM2 (E) at 5 PFU/cell in the presence of CHX, ActD, DRB, α-amanitin (α-Am; following a 1-h pretreatment of cells), or no drug as indicated for 8 h. Cell lysates were then prepared and subjected to SDS-PAGE and immunoblotting for phospho-IRF3, total IRF3, viral proteins, and actin.

The inhibitory effect of ActD on activation of the IFN induction cascade by influenza A viruses strongly suggested that viral polymerase activity was required for this process. We next determined whether IRF3 activation was also sensitive to other inhibitors of viral RNA synthesis. Alpha-amanitin inhibits both RNA polymerase II initiation and elongation (39) and thus, like ActD, prevents synthesis of viral mRNA, cRNA, and vRNA (33, 34). In contrast, the RNA polymerase II elongation inhibitor 5,6-dichloro-1-β-d-ribofuranosyl-benzimidazole (DRB) (40, 41) does not affect primary viral transcription but inhibits cRNA and vRNA synthesis (and therefore secondary viral transcription) (42) by preventing the nuclear export of viral transcripts (23, 43). Consistent with previous publications, no viral mRNA, cRNA, or vRNA synthesis can be detected in infected cells following treatment with either α-amanitin or ActD (Fig. 6A). Similarly, DRB impaired cRNA and vRNA synthesis, but viral mRNA could still be detected under DRB treatment conditions (at lower levels than in untreated cells, due to impairment of vRNA synthesis and thus secondary transcription). *In situ* hybridization experiments using a probe for NP mRNA confirmed that ActD and α-amanitin efficiently inhibited viral mRNA synthesis and that DRB treatment did not prevent mRNA synthesis but resulted in the retention of NP transcripts in the nuclei of infected cells (Fig. 6B). Previous reports indicated that DRB-mediated inhibition of mRNA nuclear export was a segment-specific effect that did not affect the export and subsequent translation of NP mRNA; here, we clearly observe both nuclear retention of NP mRNA (Fig. 6B) and efficient inhibition of NP expression by DRB at the protein level (Fig. 7), indicating that under these treatment and infection conditions DRB does inhibit NP mRNA export. These discrepancies were not due to differences in DRB concentration between this and previous studies, since we observed the same inhibition of NP expression by DRB at much lower concentrations of DRB (data not shown), but may instead reflect cell-type-specific differences.

The effects of these inhibitors on IRF3 activation by influenza virus were next examined. Consistent with data presented above, phospho-IRF3 could clearly be detected under CHX treatment conditions for cells infected with Ud, Ud-Δ99, PR8, and PR8-ΔNS1 (Fig. 7A to D). In contrast, negligible IRF3 activation by these viruses was detected in the presence of either α-amanitin or DRB, mirroring the effects of ActD treatment. Inhibition of IRF3 activation by these drugs was specific to influenza virus infections, since they had no effect on IRF3 activation by PIV5-VΔC vM2 (Fig. 7E). Clearly then, the inhibitory effects of ActD on activation of the IFN induction cascade by influenza A viruses also extend to other inhibitors of viral RNA synthesis that possess different modes of action.

**FIG 8:**
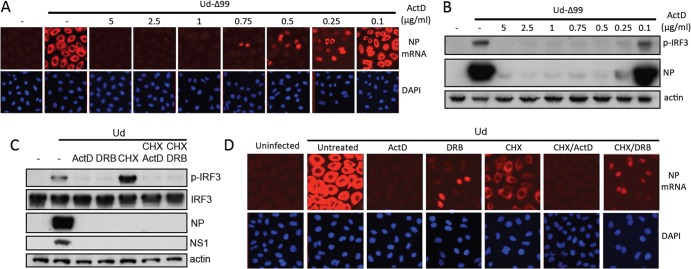
IRF3 activation by influenza viruses requires RNA synthesis and nuclear export. (A and B) A549 cells were infected with 5 PFU/cell of Ud-Δ99 for 8 h in the presence of various concentrations of ActD. (A) Viral mRNA synthesis and localization were then examined by *in situ* hybridization using an NP mRNA probe (red). Nuclei were stained with DAPI (blue). (B) Duplicate monolayers were harvested and subjected to SDS-PAGE and immunoblotting for phospho-IRF3, viral NP, and actin. (C) A549 cells were infected with Ud (5 PFU/cell) in the presence of ActD, DRB, CHX, or combinations of these drugs as indicated. After 8 h, cell lysates were prepared and subjected to SDS-PAGE and immunoblotting for phospho-IRF3, total IRF3, viral proteins, and actin. (D) Duplicate monolayers were treated as described for panel C and fixed at 8 h p.i. Viral mRNA synthesis and localization were visualized by *in situ* hybridization using a probe for NP mRNA (red). Nuclear material is stained with DAPI (blue).

That DRB, which blocks viral mRNA nuclear export but not synthesis, functioned as a potent inhibitor of viral IRF3 activation strongly suggested that both RNA synthesis and nuclear export are required for IFN induction by influenza virus under these experimental conditions. While the high concentrations of ActD used throughout this study inhibit primary viral transcription, lower ActD concentrations do not; however, like DRB treatment, low ActD concentrations limit secondary transcription and vRNA replication by preventing the nucleocytoplasmic transport of viral mRNA (23, 44). Within a concentration range of 0.25 to 0.75 μg/ml of ActD, we observe nuclear retention of NP mRNA by *in situ* hybridization; concentrations below this range do not prevent cytoplasmic accumulation of mRNAs, while mRNA synthesis is inhibited at concentrations above this range (Fig. 8A). Despite viral mRNA synthesis proceeding at 0.5 and 0.75 μg/ml, therefore, the viral mRNA is entirely nuclear in localization (Fig. 8A); at these concentrations, no IRF3 activation was detectable (Fig. 8B). Very small amounts of phospho-IRF3 are detectable at 0.25 μg/ml, which correlates with the appearance of a small proportion of cells that exhibit cytoplasmic mRNA staining and subsequent synthesis of low levels of NP protein. Further evidence for the requirement for both RNA synthesis and nuclear export for IFN induction comes from the observation that IRF3 activation induced by Ud in the presence of CHX is sensitive to ActD or DRB, which correlated with inhibition of RNA synthesis for the CHX/ActD condition and with inhibition of RNA export for the CHX/DRB condition (Fig. 8C and D). There is therefore a strong correlation between the inhibitory effects of ActD, DRB, and α-amanitin on activation of the IFN induction cascade and their ability to inhibit either viral RNA synthesis or nuclear export. Taken together, our data indicate that, in this experimental system, input viral genomes do not function as a major PAMP and suggest instead that synthesis and nuclear export of a viral RNA species (that is likely distinct from progeny vRNPs) are required for activation of the IFN induction cascade.

## DISCUSSION

The requirements for viral replication and transcription activities in the activation of the IFN induction cascade in cells infected with influenza viruses have not been comprehensively studied previously. We have addressed these questions by examining activation of the IFN induction cascade by influenza virus in the presence of inhibitors of viral RNA synthesis. Unlike previous studies, we have directly examined activation of endogenous components of the IFN induction cascade within infected A549 cells, permitting us to study the conditions under which PAMPs are generated by influenza viruses in these cells. We clearly demonstrate activation of the IFN induction cascade and transcription of IRF3-responsive genes under conditions where viral protein synthesis and therefore the generation of cRNPs and vRNPs are efficiently blocked by CHX treatment or NP siRNA. It remains possible that inhibition of NP synthesis by CHX and NP siRNA is not complete and that small amounts of NP protein are being generated but are undetectable by Western blotting. However, following these treatments we see neither full-length progeny cRNA and vRNA by primer extension analysis nor the synthesis of other viral proteins, indicating that secondary transcription is effectively blocked. As such, these experiments allow us to study the contribution of viral protein synthesis and cRNP/vRNP synthesis to IFN induction and indicate that full-length progeny genomes are not required for IFN induction in influenza virus-infected A549 cells. In complete opposition to our findings, a previous study identified progeny genomes generated during genome replication as the IFN-inducing PAMP produced by influenza virus and even demonstrated that CHX treatment of influenza virus-infected cells inhibited the accumulation of “stimulatory” RNA (11). The experimental data on which these conclusions were primarily based were a series of experiments in which RNA was extracted from reconstituted vRNPs and transfected back into cells along with an IFN-β reporter plasmid; since RNA extraction strips the associated viral nucleoprotein and polymerase from virus genomes, it would thus be expected that the resultant unencapsidated genomes (that would not normally be found in infected cells) would efficiently activate the IFN response. Moreover, during vRNA synthesis, the 5′ termini of newly synthesized progeny genomes are bound and protected by a polymerase complex distinct from the replicative polymerase (45) and should not be able to activate RIG-I. It should be stressed, however, that while genome replication was not essential for IFN induction in our experiments, we found that IFN induction by PR8-ΔNS1 was enhanced when genome replication was permitted to occur. This result may reflect the increase in numbers of templates, possibly defective genomes (discussed below), from which a PAMP can be generated when genome replication is permitted to occur. In support of this, IRF3 activation by PR8-ΔNS1 in the presence of CHX (but not in the presence of ActD) can be considerably increased by increasing the amounts of input virus (Fig. 3E). Alternatively, multiple types of PAMPs may be generated by PR8-ΔNS1, of which one or more may require genome replication.

We additionally demonstrate that viral RNA synthesis is required for activation of the IFN induction cascade by influenza viruses. In the presence of the cellular and viral transcription inhibitors ActD and α-amanitin, we observed striking inhibition of IRF3 phosphorylation and inhibition of IRF3 and p65 nuclear translocation. This inhibitory effect correlated with inhibition of viral polymerase activity since no viral RNA synthesis could be detected in the presence of these drugs. While this article was in preparation, it was reported that IFN induction by RNA viruses did not require genome replication because RIG-I is activated by incoming RNA virus nucleocapsids (46). While most of the supporting evidence came from experiments using bunyaviruses, the authors extended their conclusion to all viruses whose genomes possess a 5′ppp dsRNA panhandle structure, including influenza viruses. We demonstrate here that incoming vRNPs cannot be the predominant inducer of IFN in influenza virus-infected A549 cells, since treatment with the transcription inhibitor ActD, α-amanitin, or DRB (which does not interfere with the entry and nuclear import of incoming nucleocapsids) very effectively inhibits activation of the IFN induction cascade. Additionally, NS1, the principal IFN antagonist of influenza viruses, is not present in the virus particle and is expressed only following RNP nuclear import and transcription; if incoming vRNPs acted as the predominant RIG-I ligand, RIG-I would be activated prior to any NS1 expression and lead to IFN induction in every infected cell, a situation that we have shown does not occur (47). However, while our data rule out input vRNPs as a major PAMP in our experiments, we could still observe IRF3 and p65 nuclear translocation in small numbers (∼1%) of infected cells in the presence of ActD. While this may represent an incomplete block on viral RNA synthesis by ActD, it may also be consistent with input viral genomes constituting a minor PAMP population that can induce IFN independent of viral polymerase activity in a minority of cells. Although a simple dsRNA panhandle structure formed by base pairing between the partially complementary termini of influenza vRNAs was suggested by early studies, more-recent evidence favors a more complex “corkscrew” conformation for vRNPs involving the formation of short hairpin loops at each end of the vRNA (48–53). While limited base pairing occurs within and between the 3′ and 5′ ends in this structure, the proximal 9 or 10 nucleotides of the termini do not bind each other to form dsRNA (1, 49, 54). Moreover, the vRNA termini are obscured in the presence of polymerase (1), and despite the partial terminal complementarity of vRNA segments, vRNPs behave as single-stranded RNA in the absence of polymerase (12). The formation of the stretches of exposed dsRNA with a 5′ppp that are required for efficient RIG-I activation are thus precluded in this corkscrew structure (9, 10, 55). However, it has been reported that the 3′ and 5′ ends of naked vRNA do base pair to form dsRNA (56, 57); such a product could therefore activate RIG-I if it were generated as a result of erroneous replication.

The nuclear localization of RIG-I (or indeed any PRR) has not hitherto been shown, yet it is thought that all influenza virus RNA synthesis occurs in the nucleus. Thus, for an influenza virus RNA to function as a PAMP and induce IFN, it must presumably be exported to the cytoplasm, where it can activate RIG-I. Progeny vRNPs translocate to the cytoplasm in order to be incorporated into new virions, yet these are unlikely to be a major RIG-I agonist because we observe strong activation of the IFN induction cascade under conditions where progeny vRNA is not synthesized. That the IFN induction cascade is not activated when virus polymerase activity is inhibited suggests two possible scenarios for the generation/exposure of the PAMP. The first is that limited polymerase activity is required to displace RNA-associated proteins and expose input virus genome to RIG-I, but this is unlikely to be the case since viral polymerase activity is thought to be restricted to the nucleus and it is difficult to envisage how the input genome itself could function as an efficient PAMP for the reasons outlined above. Our data support a second scenario in which polymerase activity is required to generate an RNA product (that is distinct from progeny vRNA molecules) that translocates to the cytoplasm, where it can be recognized by RIG-I. Consistent with the latter explanation is the observation that IRF3 activation is very efficiently inhibited by DRB treatment and by low concentrations of ActD. Under these conditions, viral mRNA synthesis was not inhibited but viral transcripts were retained in the nucleus. Thus, in this situation, the PAMP would be generated by the polymerase but would be unable to translocate to the cytoplasm and would therefore be unable to activate RIG-I. The nature of this RNA species and how it would activate the IFN response is unclear. Although we have correlated activation of IRF3 with the synthesis and export of viral mRNAs, normal transcripts are capped and polyadenylated and should therefore be indistinguishable from cellular transcripts in terms of RIG-I activation; if generated during primary viral transcription, the PAMP would therefore have to be an aberrant transcription product. Unencapsidated cRNAs can be generated by the polymerase in the absence of NP (58–62) but are unstable in the absence of newly synthesized viral polymerase and NP (24); these RNAs should possess the 5′ppp required for RIG-I activation. These RNAs have been shown to be generated in the presence of both **C**HX and ActD (24, 33) and so are presumably also generated in the presence of DRB and α-amanitin. If these unencapsidated products are generated in the presence of ActD, DRB, and α-amanitin, however, they may still be retained in the nucleus (thus preventing them from potentially activating RIG-I) since these inhibitors also affect nuclear export of influenza virus RNAs (Fig. 8) (23, 44).

In conclusion, we have demonstrated that RNA synthesis and nuclear export are required for activation of the IFN induction cascade by influenza viruses and that incoming vRNPs do not therefore function as a major PAMP in this experimental system. Furthermore, we suggest that the major viral PAMP is distinct from progeny vRNPs since we observe activation of the IFN induction cascade even in situations where the generation of progeny vRNPs is inhibited. However, the exact nature of these PAMPs still needs to be characterized. Our favored explanation, which we are currently investigating, is that they are aberrant RNA products, perhaps produced by certain types of defective genomes. Intriguingly, defective viruses have been implicated in the induction of IFN by influenza A viruses previously (7, 63–65). Such a scenario would be similar to that observed for paramyxoviruses, whereby defective viruses are primarily responsible for IFN induction and can induce IFN in the absence of virus genome replication or protein synthesis (7, 19, 20, 35, 47, 66, 67). However, the precise nature of the influenza virus PAMP and the stage of the virus life cycle at which it is generated may differ depending on the type of host cell being infected. Thus, it will be of great interest to determine whether observations made in this study also extend to other cell types, such as immune cells and plasmocytoid dendritic cells, particularly given the critical role of the latter in IFN production during *in vivo* infections.
